# Optimizing Diffusive Transport Through a Synthetic Membrane Channel

**DOI:** 10.1002/adma.201203500

**Published:** 2012-11-15

**Authors:** Stefano Pagliara, Christian Schwall, Ulrich F Keyser

**Affiliations:** Cavendish Laboratory, University of CambridgeJJ Thomson Ave, Cambridge, CB3 0HE, UK

**Keywords:** membrane protein channels, channel-facilitated diffusion, holographic optical tweezers, microfluidics, colloids

Transport of ions,^1^, ^2^ proteins,^3^ antibiotics^4^, ^5^ and other macromolecular solutes through channels and pores is ubiquitous in nature. In particular channel-facilitated diffusion relies on optimized binding sites for the transported particles inside the channel.^6^ Well characterized examples include membrane channels such as maltoporins^7^, ^8^ or aquaglyceroporin^9^ found in abundance in bacterial membranes. This has been confirmed by (i) ex situ crystallographic structure studies,^10^, ^11^ (ii) indirect ionic current measurements through protein channels reconstituted into planar lipid bilayers^12–14^ and (iii) molecular dynamics simulations.^9^ These results suggest that organisms can maximize nutrient uptake driven by diffusion by favoring intimate interaction between the protein channel and the translocating species. This is counterintuitive since a strong binding site implies a long residence time in the channel. However, a few theoretical studies have independently rationalized such findings by considering the transport of particles through a channel using a continuum diffusion model based on the Smoluchowski equation,^15^ discrete stochastic models^16^, ^17^ or a generalized macroscopic Fick's diffusion law,^18^ all demonstrating that an attractive potential in the channel may enhance the particle flux. Remarkably, one intriguing approach predicts a maximum in the diffusive current with respect to the binding potential.^15^

Testing the validity of these models with an in situ tunable model system on the micro scale resembling a protein channel and its transported species would open the way to a deeper understanding of the general physical mechanism and could ultimately help in the investigation of more complicated processes such as the flux of antibiotics across bacterial membrane proteins.^19^ In order to mimic channel-facilitated membrane transport, we combine microfluidics,^20^ particle tracking^21^ and holographic optical tweezers (HOTs)^22^ to create a fully controlled and tunable environment to study passive diffusion.

Our experimental model system mimics facilitated membrane transport, using 450 nm polystyrene colloidal particles as translocating species and a microfluidic chip constituted of two macroscopic baths separated by a polydimethylsiloxane (PDMS) barrier and connected via a sub-micrometer channel^23^ as the protein channel. Since the channel cross section is close to the particle dimension we can assume quasi one-dimensional (1D) diffusion along the channel length (upper scheme in **Figure** **1**).

**Figure 1:**
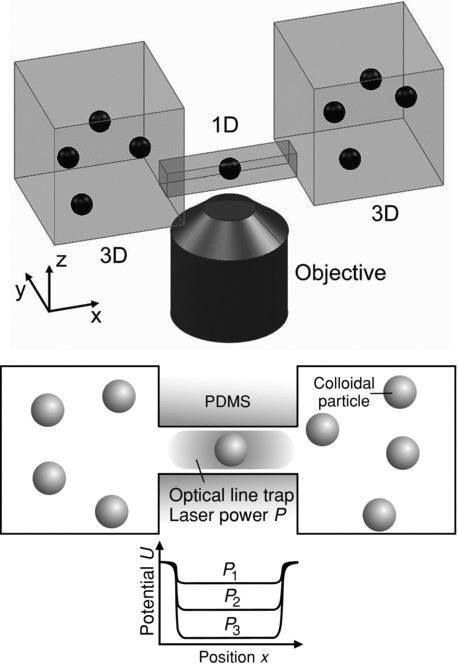
Illustration of the synthetic membrane channel: Upper panel: two macroscopic reservoirs are connected by a channel allowing for particle transport. Particle diffusion is confined along the channel mimicking quasi-1D diffusion through membrane channels. The diffusion of the particles is quantified by imaging with a 1.4 N.A. 100× objective. Lower panel: simplified top-view of the geometry shown above. Particles diffusing in the channel are subject to an attractive potential from an extended laser line trap generated by holographic optical tweezers. The laser power *P* can be used to tune the depth of the binding potential in the channel (sketch below the channel) thus mimicking channel-facilitated transport through proteins.

We mimic facilitated transport by creating in the channel a binding site for the diffusing particles with extended optical line traps generated by HOTs. Our approach offers the unique possibility to control the potential landscape without affecting the channel geometry (lower scheme in Figure 1). We use particle tracking based on digital video microscopy to measure the translocation probability, the average lifetime and the diffusion current through the channel. The single-particle occupation probability distribution along the channel length allows the determination of the potential energy generated by the optical line trap. We demonstrate that the average lifetime increases, as expected, upon coupling a line trap in the channel but at the same time the translocation probability is significantly elevated. Combined, this leads to a maximum in the diffusive current, a factor of three higher than for channels without binding potential.

A microfluidic chip was designed and fabricated through multilevel lithography^23^ to mimic biological membrane channels: a semi-cylindrical channel of length 4 μm and radius 0.75 μm is in equilibrium with two macroscopic reservoirs filled with 450 nm polystyrene particles dispersed in a 5 mM KCl solution. Particles mainly explore the regions closer to the channel entrance, up to 300 nm inside the channel (scheme in **Figure** **2** and Video 1). It is noteworthy to observe that without any line trap the channel is either empty (with a probability *p_e_* = 0.94) or populated by a single particle (*p_1_* = 0.06) while it is only rarely occupied by two or more particles at the same time (*p_>1_* = 0.001). Due to the dimensions of the channel and particle we assume 1D diffusion along the channel length. Eventually some particles reach the central part of the channel (micrograph in Figure 2 and Video 2). This can be visualized by plotting the number of times *d(x)* we find a particle at position *x* in the channel, (histogram in Figure 2, see methods for details) as measured by tracking 342 independent particle trajectories (see example trace in Video 3): the entrances of the channel are explored by the particles three times more often with respect to its central part. From our results we determine the particle concentration in the channel to be around (0.017 ± 0.011) μm^−3^ which is close to the particle concentration in the bulk (0.01 μm^−3^). Overall the average translocation probability and diffusion current are *p_Tr_* = (0.07 ± 0.05) and *J* = (7 ± 3) h^−1^, respectively. This is in very good agreement with a simple diffusion model predicting *J_Pr_ =* 6.7 h^−1^ (see Methods), using the measured bulk and channel diffusion coefficients of 0.88 μm^2^/s and 0.25 μm^2^/s, respectively, and a volumetric particle concentration of 0.01 μm^−3^.

**Figure 2:**
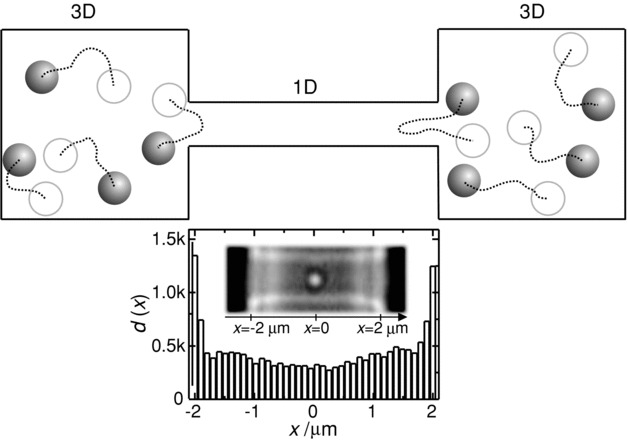
Free-diffusion in channel: Scheme: single particles enter the channel from both reservoirs, explore its outer regions and leave the channel mostly returning to the original reservoir as indicated by the trajectories (dashed lines). Graph: experimentally measured histogram of the position dependent particle distribution *d(x)* along the channel length *x*, showing that particles mainly explore the channel entrances and only rarely reach the centre of the channel. The data were extracted by analyzing the trajectories of 342 particles from nine 23 min long videos from three independent measurements. Inset: typical image of a single 450 nm colloidal particle in the central part of a typical channel with width, thickness and length of 1.2, 0.9 and 4 μm, respectively. Dashed lines indicate the edges of the channel and particle, respectively.

It was suggested^7^ that living systems enhance the transport of particles through their membrane channels by developing specific binding sites. Here, we used HOTs to create a range of potential landscapes with optical line trap of length, *λ,* fixed at 3 μm. We can easily tune the potential depth using the laser power, *P* (**Figure** **3**a), thus mimicking binding sites of different strengths. Our line trap creates an attractive potential for the diffusing particles (Figure 1). Following the diffusion over several hours, we generated histograms of the particle distribution along the channel (Figure 3b) which, in contrast to the case of free diffusion (Figure 2), leads to an increase in the particle population of the channel upon increasing laser power (top panel in Figure 3b). Importantly in the case of the highest laser power the probability to find the channel empty decreases to *p_e_* = 0.74, while finding more than one particle (*p_>1_*) increases up to 0.09.

**Figure 3:**
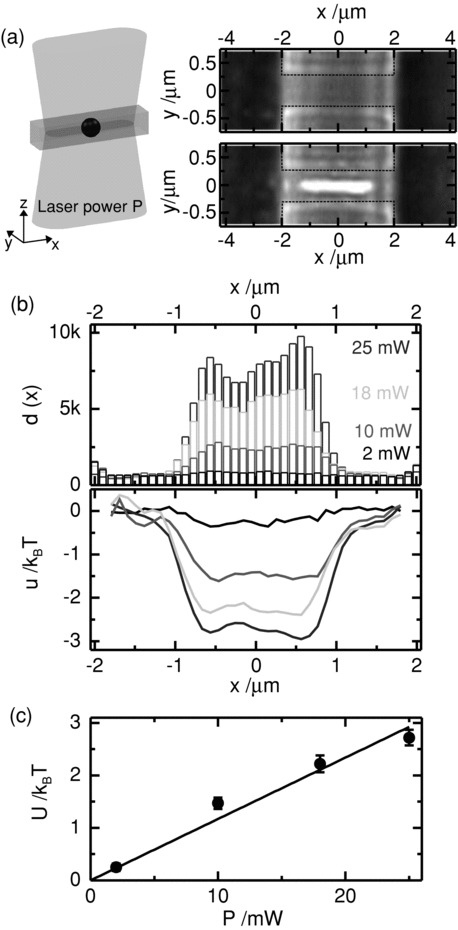
Characterization of potential in channel: (a) Scheme: a laser line trap based on holographic optical tweezers in the channel creates an attractive potential for the diffusing particles. Micrographs: light microscopy images of a channel without particles imaged with hot mirror in front of the camera (upper panel) and without (lower panel). The lower image clearly shows the extension of the IR-laser focus creating the line trap. Dashed lines indicate the channel edges. (b) Histograms of position dependent particle distribution *d(x)* along the channel for laser powers of *P =* 2, 10, 18, 25 mw and corresponding energy potential landscape *u*(x) as evaluated by using the Boltzmann distribution (see Experimetal Section). Histograms for each laser power were created by averaging over particle trajectories extracted from 9 videos, each with a length of 23 min. (c) Linear dependence of the average well depth *U* as a function of the applied laser power *P*. *U* was calculated by averaging |*u*(*x*)| for the data in (b) for the central part of the channel for -0.75 μm< *x* <0.75 μm. The solid line is a linear fit to the data forcing the intercept to 0.

Moreover while the counts in the outer regions do not significantly change, the probability to find a particle in the central part for *P =* 25 mW is more than 30 times higher. This clearly shows that the attractive potential facilitates particles reaching the centre of the channel. We use the position distribution to determine the potential profile *u(x)* by using the Boltzmann distribution (see Methods). The profiles of the trap (bottom panel in Figure 3b) show a slight increase of the effective trap length, while the average depth *U* linearly increases with the laser power, as expected (Figure 3c).^21^

The presence of the binding site forces the incoming particles to explore the channel for longer times compared to free-diffusion. This increases the translocation probability *p_Tr_* up to a value of (0.11 ± 0.02) for a potential well of depth around 1.5 k_B_T (**Figure** **4**a). At the same time, the lifetime *τ* increases with *U* (Figure 4b) as predicted by the model described by Equation (1)^24^ where *τ* is defined as the average of the time differences between the instants in which each single particle enters *t_In_* and leaves the channel *t_Out_*. For the maximum value of *U* = ∼2.7k_B_T, *τ* is around 30 s and thus 15 times larger compared to the free-diffusion regime.

**Figure 4:**
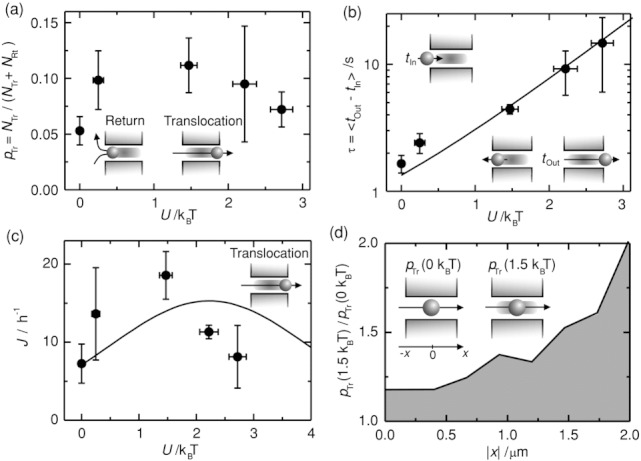
Optimal trapping potential-facilitated transport: (a) Dependence of the average translocation probability *p_Tr_* = *N_Tr_/(N_Tr_ + N_Rt_)* on the average potential depth *U*. *N_Tr_* denotes the number of particles translocating from one reservoir to the other, while *N_Rt_* is the number of particles returning to the same reservoir (see insets). (b) The average lifetime *τ* increases as a function of *U. τ* is the difference between the time the particle enters *t_In_* and leaves the channel *t_Out_* to either reservoir (see insets). Solid lines are predicted values calculated according to Equation (1) with the experimental parameters determined from our measurements. (c) The flux of particles translocating (see sketch) the channel per hour *J* as a function of *U* shows a clear maximum. *J* is increased by a factor of three at optimum *U = ∼*1.5 k_B_T. The solid line is a prediction calculated using Equation (2) and the experimental parameters (see Experimental Section). (d) Dependence of the ratio between the probability to translocate a channel for the optimal potential *p_Tr_*(*U =* 1.5 k_B_T) and the one to translocate a channel in free-diffusion *p_Tr_*(*U =* 0) as a function of the particle position in the channel |*x*|. This illustrates that the line trap increases the translocation probability for particles at the opposite entrance. Error bars are calculated as the standard deviation of the corresponding measured values in three independent experiments for the vertical axes and as in Figure 3c for the horizontal axes.

We can not only measure the potential depth, the translocation probability and the lifetime but follow each particle individually. This allows for determining the diffusion current *J* as a function of *U* as shown in Figure 4c. This indicates a clear maximum: *J* increases up to an average value of 18 h^−1^ for *U* = 1.5 k_B_T and then decreases to 7 per hour for the deepest well with *U =* 2.7 k_B_T (Figure 4c). Even for our simple attractive potential, there is an optimal *U* enhancing *J* by a factor of three. The maximum is explained by considering that at *U =* 0, particles attempt to enter but are not likely to reach the centre of the channel, while at 0 < *U <* 1.5 k_B_T, the channel is populated but *τ* is only slightly increased, leading to an enhanced transport. However, for *U >* 1.5 k_B_T, the particles are trapped in the channel for longer times effectively decreasing *J*. Berezhkovskii and Bezrukov^15^ developed a model for *J* (see Equation (2)) which predicts a maximum in very good agreement with our data (solid line in Figure 4c).

The increase in *J* is a consequence of the change in the translocation probability, *p_Tr_*(|*x*|, *U*). Figure 4d shows *p_Tr_*(*U =* 1.5 k_B_T*)/p_Tr_*(*U =* 0) as a function of position |*x*|*.* For *U* = 0 particles will escape to the original reservoir in 75% of the cases, while at *U* = 1.5 k_B_T more particles reach the channel centre at |*x*| *=* 0 and may translocate.

The presence of one particle in the channel does not exclude other particles from entering. Figure 4 shows our complete experimental results allowing the presence of more than one particle. The model assumes that only a single particle can be in the channel. For this case, we extract *p_Tr_* by ignoring any further particle attempting to enter. We find that *p_Tr_* increases up to a value of 0.11 for the deepest potential well as expected while the dependence of diffusion current and lifetime remains unchanged (see Figure S1 in Supporting Information).

We have shown here that optimizing the transport through a channel is possible using a tunable binding potential. Our experimental model system sheds light on the general physical principles governing diffusive currents and suggests a pathway to enhance particle flux. This generic finding in our simplified model system suggests a pathway to a deeper understanding of more complicated mechanisms ongoing in living systems such as drug uptake where molecular interactions and solvent properties play a major role.^4^, ^5^

The wide range of available colloidal particles, in conjunction with the presented experimental approach will allow for testing a variety of transport processes through quasi 1D structures^25^, ^26^ relevant for catalysis, osmosis and particle separation^27^, ^28^ or systems based on entropic barriers to control transport.^29^ Other relevant examples are the recently introduced nanopores mimicking transport through the nuclear pore complex.^30–32^

In summary we presented a synthetic mimic of facilitated membrane transport by exploiting building blocks such as colloidal particles, microfluidics equipped with a sub-micrometer channel and holographic optical tweezers. We characterized the particle exchange between the bulk and the channel in terms of translocation probability, lifetime and particle flux. We mimicked the interaction between the transported species and the channel by coupling an optical line trap and mapped the energy potential by measuring the position dependent probability distribution. The average lifetime and the translocation probability increase in the presence of the optical line trap indicating that diffusive flux through channels can be enhanced with an optimized potential. Our approach opens the way for improving the design of biological and synthetic nanochannels or -pores for optimized transport.

## Experimental Section

*Diffusion model*: A single particle can be exchanged between the bulk and the channel (Figure 1) and once in the channel 1D diffusion is assumed along the channel axis. The interaction of a single particle with the channel is described in terms of a potential of mean force *u*(*x*) which acts on the particle at the point *x*.^24^ Assuming that such a potential is described by a square well of depth *U* and length *λ*, the translocation probability *p_Tr_* and the average lifetime *τ* can be predicted as in Equation (1):^24^, ^33^

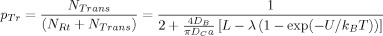


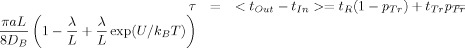
(1)
where *N_Rt_* is the number of particles failing and *N_Tr_* succeeding in the channel translocation, while *t_Rt_* and *t_Tr_* are the average return and translocation time, respectively, *D_B_* is the particle diffusion coefficient in the bulk and *D_C_* in the channel, *a* denotes the channel radius and *c* is the particle concentration. The corresponding flux of particles per second between the two reservoirs through the channel is given by Equation (2):^15^

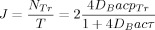
(2)
where *T* is the total measurement time and the factor 2 takes into account particle flux from both reservoirs.

*Microfluidic chip*: Details about the fabrication of our microfluidic chips equipped with sub-micrometer channels are reported elsewhere.^23^ Briefly a Platinum wire was deposited on a Silicon substrate via focused ion beam. The wire cross section was measured *in situ* by slicing the wire at one end, tilting the sample at 63° and imaging via the electron beam. The resulting profile was semi-elliptical with major (wire thickness) and minor (wire half width) axes measuring 0.9 and 0.6 μm, respectively. The profile was approximated as a semi-cylinder of radius 0.75 μm in the diffusion model predictions. Conventional photolithography, replica molding and PDMS bonding to a glass slide were carried out to define 16-μm thick reservoirs separated by a PDMS barrier and connected by (i) the channel obtained as a negative replica of the Platinum wire and (ii) two lateral channels with width and thickness of 100 and 16 μm, respectively, to facilitate hydrostatic pressure equilibration between the two reservoirs.

A slight variation in the cross section at the channel entrances may be attributed to (i) overexposure, (ii) underdevelopment of the photoresist layer and (iii) mechanical stresses induced on the elastomeric structures during the peeling-off from the mold^34^ and may explain the peaks at the channel entrances^35^ in the particle distribution histogram (Figure 2).

*Holographic optical tweezers*: An Ytterbium fiber laser (YLM-5-1064-LP, IPG Photonics) was passed through a beam expander and directed towards a phase-only spatial light modulator (SLM, LCOS X10468, Hamamatsu). The SLM was located in the Fourier domain of a 4f configuration of lenses^22^ and the beam coupled into an oil immersion objective (100×, 1.4 N.A., UPLSAPO, Olympus). Illumination was provided from above by an LED light (Thorlabs MWLED). The transmitted light was collected by the objective and reflected towards a charge coupled device (CCD) camera (The Imaging Source DMK 31BF03) by a dichroic mirror, with hot mirrors filtering out the laser light reflected by the objective. The optical line trap was generated and controlled through the SLM by using a custom-made program based on Labview (National Instruments) that implements a modified lens and grating algorithm.

*Experiment automation***:** Experiments were automated by using a custom-made program based on Labview for positioning, laser control and video acquisition and performed overnight in order to reduce the noise level. A xyz-Nanopositioning Piezo (P-561.3CD) controlled via a Digital-Multi-Channel Piezo Controller (E-725.3CD) in combination with a custom-written Labview routine based on a look up table based search and modified variance algorithm^36^ was employed to accurately find the correct focal plane in the beginning of the experiment and then keep it for the duration of the whole experiments (several hours). Briefly a look up table of images acquired at 7 different focal planes of the same feature in the chip were produced by averaging over 3 different images of the same focal plane which were acquired by scanning the sample along *z* (piezo stage steps of 50 nm). During the experiment a test image is produced every 10 seconds by averaging 3 images in the actual focal plane and compared to the images in the look up table through the average intensity variance, *σ^2^* as described in Equation (3):

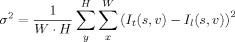
(3)
where *W* and *H* are width and height of the image, (*s*,*v*) the pixel coordinates in the image, *I*_t_(s,v) and *I*_l_(*s*,*v*) the grey scale intensities of the pixel at (*s*,*v*) in the test and look up table image, respectively. The image in the look up table that minimizes the average intensity variance determines the focal planes closest to the actual one and thus the correction needed to bring the sample in the best focal plane through the piezo stage. A similar approach was used to keep the sample fixed in the *x*-*y* plane. The laser power was cyclically varied in time through a NI DAQ Card (NI BNC-2110) managed by the same Labview programme.

*Digital video microscopy and data analysis***:** Particle tracking was realized through digital video microscopy and an off-line custom-made program based on Labview. Each single frame was processed by using a high-pass filter in the frequency domain and a threshold in order to attenuate the noise from the channel walls but not the signal from the transported particle thus enhancing the signal/noise ratio by one order of magnitude. Particle center of mass was measured through a centroid approach by using a specific Labview virtual instrument while the *x* and *y* projections of the particle trajectories were built through a nearest neighbor approach to link measurements in successive frames. Four different classes of events were defined: return to left (right) when a particle was exchanged between the channel and the left (right) reservoir and translocation from left (right) when the particle was exchanged between the left (right) and right (left) reservoir upon translocating the channel. For each event we measured the *x* and *y* coordinates every 30 ms (which is the camera frame rate) and calculated the lifetime, the space explored in the channel and the mean square displacement (MSD). Specifically The *MSD* for each single particle was measured as in Equation (4):^37^

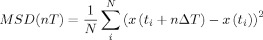
(4)
where *x(t_i_)* is the measured particle position at time *t_i_* after the entering into the channel, Δ*T* is the minimum time step between consecutive positions and was chosen to be 33 ms which is the inverse of the video acquisition frame rate, *n* is an integer multiple of the interval step *ΔT*, *N* is the number of positions which have a time difference of *nΔT*.

The diffusion coefficient in the channel was determined by fitting the *MSD* data using the Einstein relation in one dimension as in Equation (5):

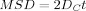
(5)

Events shorter than 2s were not included in the calculation of *D_c_* since the theoretically expected statistical variance due to the stochastic nature of the dynamics was too large. The resulting upper limit of the relative error for the diffusion coefficient (*D_rel,error_*) was estimated as in Equation (6):

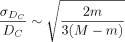
(6)
where *M* is the maximum *n* value and *m* is the maximum *n* value in the range selected in the *MSD* fit to determine *D_C_*. *m* was kept to a constant value of 10 with a corresponding error of 37% for the shortest events (*N* = 60).

We performed three independent experiments with three different microfluidic chips replicated from the same master. For each experiment we performed three cycles of measurements by acquiring a video of duration 23 minutes for each of the following coupled powers: 0, 2, 10, 18 and 25 mW.

The histograms of the position dependent particle distribution, *d*(*x*), and probability, *p*(*x*), were measured by binning the channel length, each bin corresponding to 2 pixels on the CCD camera (roughly 100 nm in the real system) and summing up the number of *x* measurements falling in each bin for all the events in all the experiments for each laser power. The potential energy profile *u*(*x*) generated by the line trap was determined by considering that it is related to the probability to find a particle within distance *dx*, *p*(*x*), through the Boltzmann distribution:^21^

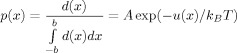
(7)
where *b =* 2 μm and the constant *A* was evaluated in the region of the channel not permeated by the optical trap upon discarding the channel outer regions where the probability distribution is not flat. Thus we calculated the average probability between the 3^rd^, 4^th^ and 5^th^ bin from left and right and evaluated the following values for *lnA*: (-3.7 ± 0.1), (-4.6 ± 0.2), (-5.2 ± 0.3) and (5.7 ± 0.2) for 2, 10, 18 and 25 mW, respectively. The average well depth, *U*, and relative error, *σ_U_*, was evaluated by averaging the potential |*u*(*x*)| for -0.75 μm < *x* < 0.75 μm and taking the standard deviation as the error.

Average lifetime, translocation probability and translocation counts were evaluated by averaging over the corresponding values measured in the three different experiments.

Finally the position dependent translocation probability was evaluated by binning the channel length as above and dividing the counts of events in which a particle had explored at least once a specific bin by the number of translocations for each coupled power.
