# Supplementary material for: Optimizing Diffusive Transport Through a Synthetic Membrane Channel
Source: Adv Mater. 2012 Nov 15;25(6):844–9. doi: 10.1002/adma.201203500 (PMC3594580; doi:10.1002/adma.201203500)
Supplement: Supplementary file 4 [file adma0025-0844-sd4.pdf]

Copyright WILEY-VCH Verlag GmbH & Co. KGaA, 69469 Weinheim, Germany, 2012.

ADVANCED MATERIALS

# Supporting Information

for *Adv. Mater.*, DOI: 10.1002/adma.201203500

# Optimizing Diffusive Transport Through a Synthetic Membrane Channel

*Stefano Pagliara, Christian Schwall, and Ulrich F. Keyser **

Submitted to ADVANCED MATERIALS

DOI: adma.201203500

**Optimizing diffusive transport through a synthetic membrane channel**

Supporting Information

By *Stefano Pagliara*, *Christian Schwall* and *Ulrich F. Keyser**

[*] Dr. Ulrich F. Keyser, Dr. Stefano Pagliara, Christian Schwall
Cavendish Laboratory, University of Cambridge, JJ Thomson Ave
Cambridge, CB3 0HE, UK

E-mail: ufk20@cam.ac.uk

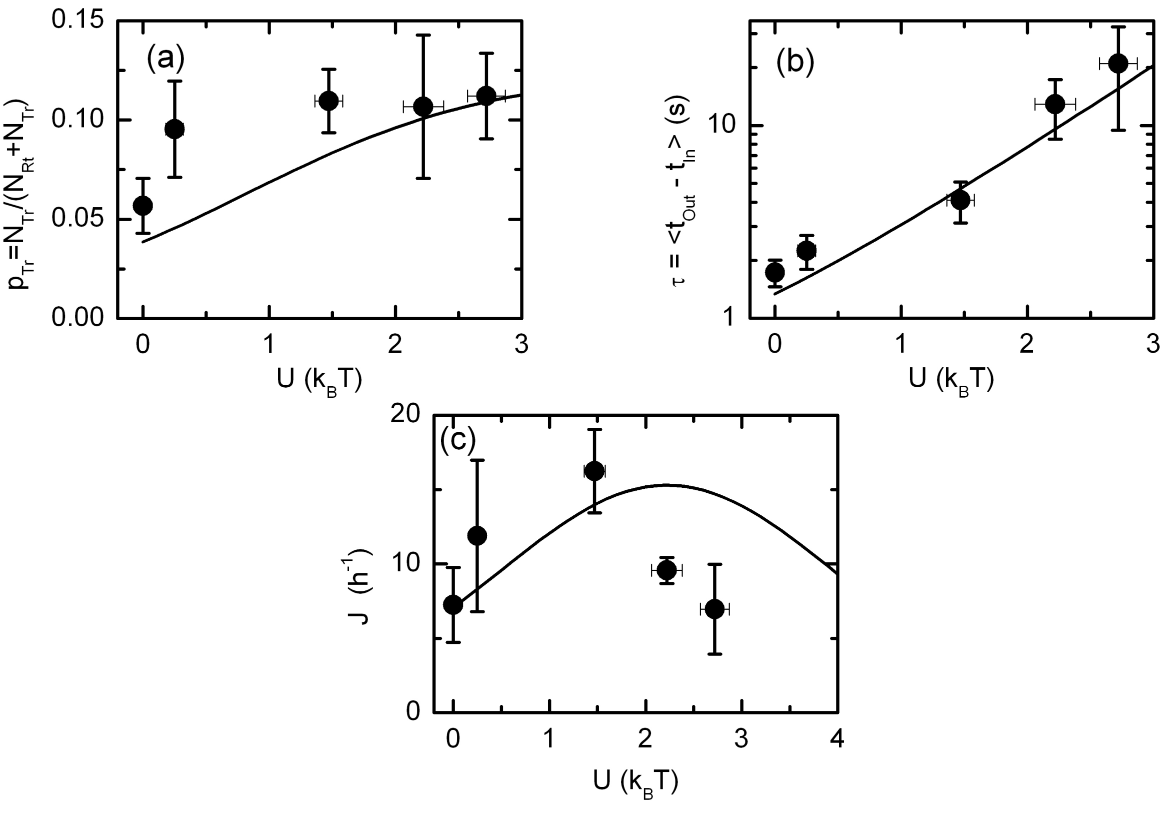


**Figure S1** Dependence of the translocation probability $p_{Tr}$ (a), lifetime $\tau$ (b) and diffusion current $J$ (c) with respect to the average potential well depth as measured by considering the channel blocked when one particle occupies it. In case the channel is occupied we do not count further particles entering.
